# Driving patterns in older adults with glaucoma

**DOI:** 10.1186/1471-2415-13-4

**Published:** 2013-02-21

**Authors:** Suzanne W van Landingham, Chad Hochberg, Robert W Massof, Emilie Chan, David S Friedman, Pradeep Y Ramulu

**Affiliations:** 1 Wilmer Eye Institute, Johns Hopkins University, 600 N. Wolfe St., Baltimore, MD, 21287, USA

## Abstract

**Background:**

The ability to drive is important for ensuring quality of life for many older adults. Glaucoma is prevalent in this age group and may affect driving. The purpose of this study is to determine if glaucoma and glaucomatous visual field (VF) loss are associated with driving cessation, limitations, and deference to another driver in older adults.

**Methods:**

Cross-sectional study. Eighty-one glaucoma subjects and 58 glaucoma suspect controls between age 60 and 80 reported if they had ceased driving, limited their driving in various ways, or preferred another to drive.

**Results:**

Twenty-three percent of glaucoma subjects and 6.9% of suspects had ceased driving (p = 0.01). Glaucoma subjects also had more driving limitations than suspects (2.0 vs. 1.1, p = 0.007). In multivariable models, driving cessation was more likely for glaucoma subjects as compared to suspects (OR = 4.0; 95% CI = 1.1-14.7; p = 0.03). The odds of driving cessation doubled with each 5 decibel (dB) decrement in the better-eye VF mean deviation (MD) (OR = 2.0; 95% CI = 1.4-2.9; p < 0.001). Glaucoma subjects were also more likely than suspects to report a greater number of driving limitations (OR = 4.7; 95% CI = 1.3-16.8; p = 0.02). The likelihood of reporting more limitations increased with the VF loss severity (OR = 1.6 per 5 dB decrement in the better-eye VF MD; 95% CI = 1.1-2.4; p = 0.02). Neither glaucoma nor VF MD was associated with other driver preference (p > 0.1 for both).

**Conclusions:**

Glaucoma and glaucomatous VF loss are associated with greater likelihood of driving cessation and greater limitation of driving in the elderly. Further prospective study is merited to assess when and why people with glaucoma change their driving habits, and to determine if their observed self-regulation of driving is adequate to ensure safety.

## Background

Over 60 million people worldwide are affected by glaucoma, a number that will increase substantially as the population ages [1]. Glaucoma prevalence is highest among the elderly, and elderly individuals with glaucoma are more likely to be visually disabled because of more advanced visual field (VF) loss and other age-related factors [2].

Driving is highly valued by older adults because it is often required for independence [3]. Furthermore, driving cessation is associated with incident depression and increased risk of entry into a long term care facility, even after controlling for demographic and health variables [3]. Indeed, the ability to travel outside the home is consistently ranked as one of the two most important visual functions by people with glaucoma [4,5].

Drivers with glaucoma perceive greater difficulty with driving, and perceived difficulty increases with severity of VF loss [6]. Drivers with glaucoma have also been shown to make more driving errors during driving simulator and on-road evaluation of driving [7,8]. Some studies have also shown that VF loss severity is associated with increased motor vehicle accidents (MVAs) [9-11], though others have shown that a glaucoma diagnosis is not associated with more accidents [12,13].

One explanation for why glaucoma does not consistently increase accident risk in all studied populations is that individuals with more advanced disease may limit or stop driving [12,14-16]. Here, we examine how driving patterns (driving limitation, driving cessation, and other driver preference) differ in glaucoma patients across a range of VF loss severities.

## Methods

### Subjects

This is a cross-sectional study of subjects recruited from the Glaucoma Clinic at the Wilmer Eye Institute of Johns Hopkins Hospital between July 2009 and June 2011. Subjects’ charts were prescreened for eligibility. Eligible subjects had to be between the ages of 60–80 years and be former or current drivers.

Glaucoma subjects were restricted to those having a physician diagnosis of primary open angle glaucoma, primary angle closure glaucoma, pseudoexfoliation glaucoma, or pigment dispersion glaucoma. Most glaucoma subjects had 24–2 VF tests within the 15 months prior to enrollment from a Humphrey Field Analyzer II (Carl Zeiss Meditec, Dublin CA) using the Standard Swedish Interactive Testing Algorithm showing a VF MD worse than −3 dB and a borderline or abnormal GHT result in both eyes. Individuals whose most recent fields were 10–2 VFs were also included, in which case their better-eye MD was defined using the last recorded 24–2 VFs.

Glaucoma suspect controls were recruited from patients with a chart diagnosis of ocular hypertension or glaucoma suspect. They were required to have a presenting visual acuity of 20/40 or better in both eyes and 24–2 VF tests within the 15 months prior to enrollment indicating a mean deviation (MD) better than −5 decibels (dB) in both eyes and normal or borderline glaucoma hemifield test (GHT) results.

This study was approved by the Johns Hopkins Institutional Review Board. All subjects gave informed consent.

### Evaluation of driving habits

Driving habits were evaluated with an interviewer-administered questionnaire taken from the Salisbury Eye Evaluation Driving Study (SEEDS), which added additional questions to other questionnaires previously used in the Salisbury Eye Evaluation [17,18]. Subjects were asked, “Have you driven a car in the past three months?” to assess driving cessation. In those who were currently driving, 9 different driving limitations were assessed: (1) not driving outside of the mid-Atlantic region (defined for this Baltimore-based study as Maryland, Virginia, Delaware, the District of Columbia and Pennsylvania), (2) not driving more than one hour away from home, (3) not driving to neighboring towns or areas, (4) not driving beyond the neighborhood, (5) not driving in the rain, (6) not driving at night, (7) not driving in unfamiliar areas, (8) driving less than twice per week, and (9) driving less than 5,000 miles (the standard for restricted driving in Maryland). Each of these limitations was assessed for the past year except for driving at night and driving in unfamiliar areas, which were assessed for the past three months. The questionnaire asked whether a person had performed a particular driving activity *at all* during the appointed time frame, not if they avoided that particular driving activity or if they were legally prohibited from doing it.

Driver preference was assessed by asking subjects, “in a typical week when you travel in a car, how often are you the driver?” Subjects who reported that they were the driver 50% or less of the time they rode in a car were considered to prefer another driver.

### Measurement of vision and covariates

Monocular visual acuities were measured using the Early Treatment Diabetic Retinopathy Study (ETDRS) chart transilluminated at 130 candelas/m^2^ and converted to the logarithm of the minimum angle of resolution (logMAR) for use in statistical analysis [19]. VF MD results were extracted from the chart. VF and visual acuity of the better-seeing eye were used for further analysis. Binocular contrast sensitivity (CS) was measured using the Pelli-Robson chart at 1 meter with subjects wearing their usual correction and converted into log units for analysis [20].

Both eyes were examined after pupillary dilation for significant lenticular changes defined as nuclear sclerosis greater than grade 2 on the Wilmer Cataract Grading system [21], blocked retroillumination in ≥4/16 of the pupil due to cortical changes, any opacity in the central 3 mm of the posterior capsule, or, in pseudophakic eyes, posterior capsular opacification (PCO) demonstrating changes more severe than the “mild” image in Findl et al [22].

Demographic information collected included age, gender, race, employment status, years of education completed, living situation (if the subject lives with any other adults), and marital status, all by self-report. Cognitive ability was assessed using the Mini Mental Status Exam (MMSE) for the Visually Impaired [23]. Depressive symptoms were detected using the Geriatric Depression Scale Short Form, with subjects demonstrating 6 or more positive responses considered to have depressive symptoms [24]. Medical comorbidities were assessed using a standardized structured medical history questionnaire and summarized as the number of comorbid conditions present [25]. We inquired about arthritis, broken or fractured hip, back problems, heart attack/myocardial infarction, angina/chest pain, congestive heart failure, peripheral vascular disease, hypertension, diabetes, emphysema, asthma, stroke, Parkinson’s, cancer (other than skin cancer), and vertigo/Meniere’s.

### Statistical analysis

Group differences for continuous variables were evaluated using the Wilcoxon rank-sum test. Chi-square analysis was used to assess differences in categorical variables (Stata 11.2, College Station, TX).

Binary outcomes, including driving cessation, driver preference, and the presence of each specific driving limitation were assessed using univariate and multivariable logistic regression models. The overall number of limitations was assessed in univariate and multivariable ordinal logistic regression models after trifurcating the number of driving limitations into three categories (<3, 3–4, and >4 limitations). These categories were chosen to fulfill the proportional odds assumption, which was verified using the Brant test.

## Results

One hundred and thirty-nine current or previous drivers participated in this study, including 81 with glaucoma and 58 suspects. Suspects and glaucoma subjects were similar with regards to most health and demographic characteristics (Table 1). Glaucoma subjects had worse visual acuity (median logMAR of 0.15 versus 0.08 in the better-seeing eye), worse VF results (better-eye median VF MD of −7.9 versus +0.2 dB), worse contrast sensitivity (1.6 versus 1.9 log units), and were more likely to be non-white when compared to suspects (Table 1).

**Table 1:** Characteristics of study participants by glaucoma status

	**Glaucoma suspect controls (n = 58)**	**Glaucoma (n = 81)**
**Vision**		
Better eye visual field, MD (dB)	0.2 (−0.7, 0.9)	−7.9* (−15.4, -4.8)
Binocular CS, log units	1.9 (1.8, 2.0)	1.6* (1.4, 1.8)
Better eye acuity, logMAR	0.08 (0.00, 0.16)	0.15* (0.08, 0.32)
Sig. cataract/PCO, either eye (%)	22.2	35.8
Sig. cataract/PCO, both eyes (%)	9.3	11.1
**Demographics**		
Age (years)	69.8 (65.6, 73.0)	70.3 (66.4, 74.5)
White race (%)	77.6	62.5*
Female gender (%)	60.3	51.3
Education (years)	17 (14, 17)	16 (14, 17)
Unemployment (%)	60.3	57.5
Lives with others (%)	81.0	81.2
Married (%)	67.2	62.5
**Health/cognition**		
MMSE-VI score	21 (20, 22)	21 (20, 22)
Comorbid illnesses (#)	2 (1, 3)	2 (1, 3)
Depressive symptoms (%)	5.2	5.9

Continuous variables reported as median (inter-quartile range).

*P < 0.05 as compared to glaucoma suspect controls.

LogMAR = logarithm of the minimum angle of resolution; MD = mean deviation; dB = decibels; CS = contrast sensitivity; PCO = Posterior capsular opacification; MMSE VI = Mini-Mental Status.

In unadjusted analyses, more glaucoma subjects than suspects had ceased driving (22.5% vs. 6.9%, p = 0.02). Glaucoma subjects also had a greater mean number of driving limitations (2.0 vs. 1.1, p = 0.004) and were more likely to have ceased driving at night compared to suspects (27.4% vs. 7.4%, p = 0.005) (Table 2). Differences between the two groups in the prevalence of all other limitations were not statistically significant, although a higher proportion of subjects with glaucoma endorsed each limitation.

**Table 2:** Driving limitations in current drivers with and without glaucoma

**Driving limitation**	**Glaucoma suspects % (n = 54)**	**Glaucoma% (n = 62)**	***P *value**
Has not driven at night*	7	27	0.005
Has not driven in the rain	0	7	0.06
Has not driven in unfamiliar areas*	19	31	0.13
Has not driven more than one hour away	15	25	0.21
Has not driven beyond the neighborhood	0	2	0.35
Has not driven to neighboring towns or areas	2	7	0.23
Has not driven outside the region	53	71	0.05
Drives <2 times per week	2	5	0.38
Drove <5,000 miles per year	15	24	0.21

*These questions refer to the past three months. All other limitations refer to the past year.

All percentages rounded to integers.

**Figure 1:**
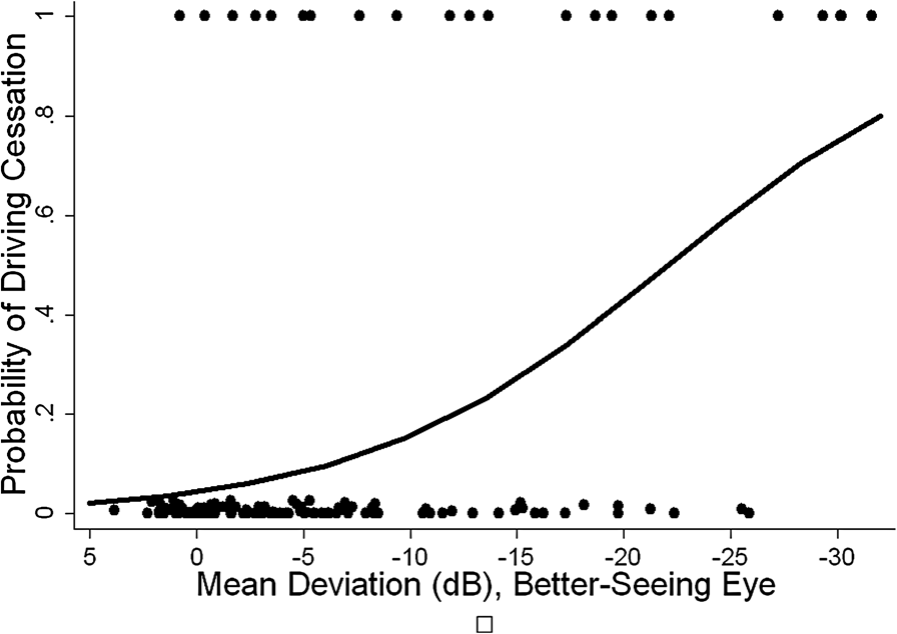
**Modeled probability of not driving as a function of better-eye visual field loss in glaucoma patients.** In addition to better-eye mean deviation, our multivariable logistic regression model includes age, gender, unemployment, cognition, comorbidities, and depressive symptoms. dB = decibels.

**Figure 2:**
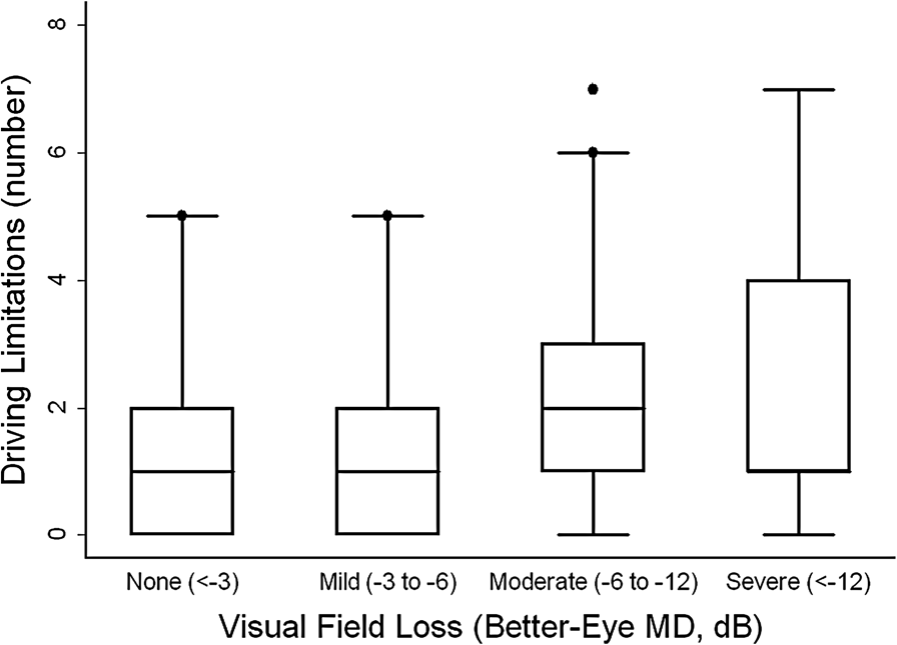
**Number of driving limitations by severity of better-eye visual field loss.** Upper and lower limits of box reflect the 75th and 25th percentile values. Median values are shown by the horizontal line within the box, and is not seen for the severe glaucoma group as the median value is the same as the 75th percentile. MD = mean deviation, dB = decibels.

When adjusting for age, race, gender, unemployment, cognition, comorbidities, and depressive symptoms, subjects with glaucoma were four times more likely than glaucoma suspect controls to have ceased driving (odds ratio [OR] = 4.0; 95% CI = 1.1-14.7; p = 0.03) (Table 3). Among glaucoma subjects, driving cessation became more likely with more severe VF loss (OR = 2.0 for each 5 dB decrement in the better-eye MD; 95% CI = 1.4-2.9; p < 0.001) (Figure 1). Glaucoma subjects were also likely to report more driving limitations than glaucoma suspect controls (OR = 4.7; 95% CI = 1.3-16.8; p = 0.02). The likelihood of reporting more limitations increased with the severity of VF loss (OR = 1.6 for each 5 dB decrement in the better-eye MD; 95% CI = 1.1-2.4; p = 0.02) (Figure 2). Significant non-visual factors associated with driving habits included a higher risk of driving cessation in subjects with depressive symptoms (OR = 16.5; 95% CI = 2.2-123.7; p = 0.01) and a greater number of driving limitations in female subjects (OR = 8.3; 95% CI = 2.0-35.2; p = 0.004).

**Table 3:** Effect of glaucoma and glaucoma severity on driving status, multivariable analysis

**Variable**	**Interval**	**Not driving**	**Increased limitations**^**§**^	**Pref. another driver**^**§**^
		***Odds ratio (95% CI)***	***Odds ratio (95% CI)***	***Odds ratio (95% CI)***
**Vision**				
Glaucoma	Present	4.0* (1.1-14.7)	4.7* (1.3-16.8)	1.7 (0.54-5.3)
Better eye visual field, MD	5 dB worse	2.0* (1.4-2.9)	1.6* (1.1-2.4)	1.3 (0.88-2.0)
Binocular contrast sensitivity	1 letter worse^+^	1.3* (1.2-1.4)	1.2* (1.1-1.3)	1.1 (0.92-1.2)
Better eye acuity, logMAR	1 line worse^++^	1.5* (1.2-1.8)	2.1* (1.3-3.5)	0.92 (0.67-1.2)
**Demographics**				
Age	5 yrs older	1.2 (0.69-2.0)	1.7 (0.96-3.1)	1.2* (1.0-1.3)
Race	White	0.6 (0.20-1.8)	1.0 (0.31-3.5)	1.2 (0.34-4.0)
Gender	Female	1.3 (0.45-3.7)	8.3* (2.0-35.2)	24.4* (5.0-11.8)
Unemployment	Present	0.7 (0.23-2.2)	3.4 (0.88-13.0)	3.0 (0.86-10.8)
Living situation	Lives with others	-	-	48.4*(1.9-1201)
Marital Status	Married	-	-	0.91 (0.46-1.8)
**Health/cognition**				
MMSE VI score	5 points lower	4.1 (0.85-19.5)	0.22 (0.02-2.6)	19.7* (1.7-227)
Comorbidities	1 illness	1.0 (0.72-1.4)	1.20 (0.84-1.7)	-
Depressive Symptoms	Present	16.5* (2.2-123.7)	0.43 (0.03-5.7)	-

Odds ratios for vision variables were each derived from separate multivariable models including all non-visual covariates shown.

Odds ratios for non-vision variables were all derived from multivariable models including better-eye MD and all non-visual variables shown.

^**§**^Refers to current drivers only.

^+^Corresponds to 0.05 log unit change.

^++^Corresponds to 0.1 logMAR change.

*P < 0.05

Additional multivariable regression models adjusting for the same characteristics described above compared the relative impact of different elements of vision (VF, visual acuity, contrast sensitivity, and presence of cataract) on driving cessation and limitations. In models that included both VF MD and visual acuity, the associations of VF loss with both driving cessation (OR = 1.7; 95% CI = 1.1-2.5; p = 0.008) and driving limitation (OR = 1.6; 95% CI = 1.0-2.4; p = 0.04) persisted, as did the association between visual acuity and driving cessation (OR = 1.3; 95% CI = 1.0-1.6; p = 0.03). In models including both better-eye VF MD and contrast sensitivity, only the association between contrast sensitivity and driving cessation (OR = 1.3; 95% CI 1.1-1.6; p = 0.002) remained significant, though the two measures of vision loss were significantly correlated (r = 0.75; p < 0.001). Finally, in models including both cataract/PCO and better-eye VF MD, the associations between glaucoma severity and driving cessation (OR = 2.0; 95% CI = 1.4-2.9; p < 0.001) and driving limitations were unchanged (OR = 1.6; 95% CI = 1.1-2.4; p = 0.02).

Driver preference was not associated with glaucoma or VF loss severity, even when adjusting for age, race, gender, employment, living situation, marital status, and cognition (Table 3). Preferring another driver was dramatically more likely in females (OR = 24.4; 95% CI = 5.0-118; p < 0.001), in those who live with another adult (OR = 46.2; 95% CI = 1.9-1094; p = 0.02), and in those with lower cognitive ability (OR = 19.8; 95% CI = 1.7-227; p = 0.02). No other characteristics predicted preferring another driver (P > 0.05).

## Discussion and conclusions

Individuals with glaucoma were significantly more likely than glaucoma suspects to have limited or stopped their driving in this clinic-based study. Driving cessation and driving limitations were also more common with more severe VF loss (Figures 1 and 2).

The presence of the association between glaucoma and driving cessation found in the current work corroborates previous studies. The Blue Mountain Eye Study reported a greater than 2-fold increased odds of driving cessation for subjects with glaucoma or impaired visual acuity after adjusting for age and gender [16], though subjects were not classified by disease severity. The Salisbury Eye Evaluation found an association between bilateral glaucoma and driving cessation in the elderly and a 2-fold greater risk of driving cessation with every 5 dB decrement in the better-eye VF [14]. The consistency of findings across studies supports the idea that driving cessation becomes more common with greater VF loss in numerous driving environments.

Previous studies have differed in their assessment of whether people with glaucoma are more likely to limit their driving. The Salisbury Eye Evaluation found no significant associations between glaucoma and driving limitations, but our study and two others did [12,14,15]. The discrepancies between different studies may be explained by demographic differences in the study populations. For example, our study population was younger (mean age 70) and our study location was urban while the Salisbury Eye Evaluation assessed an older (mean age 80), rural group. The current study is the first to demonstrate greater driving limitations with increasing disease severity.

Driving cessation is clearly an effective method for avoiding some of the risks associated with VF loss. However, the extent to which limitation of driving is a successful method for balancing safety and independence remains unknown. One possibility is that those who limit their driving due to VF loss succeed in reducing their risk of MVAs to a level similar to or even lower than other drivers. This hypothesis could partially explain why some previous studies found that having a glaucoma diagnosis may not increase older individuals’ risk of MVAs [12,13]. However, a second possibility is that, despite limiting their driving, individuals with glaucomatous VF loss still drive poorly and/or unsafely. This is supported by studies showing that VF loss is a risk factor for MVAs [9-11]. Further study is needed to clarify the adequacy of these self-imposed driving limitations in keeping drivers safe.

Another adaptation that drivers with glaucoma may make is deferring to another driver. However, neither glaucoma nor the severity of VF loss predicted driver preference in the current study, which implies that glaucoma is not a factor influencing driver preference independent of other more significant factors such as gender and age. As such, the selection of a driver within a family may often be determined by traditional gender roles rather than by driving skill.

Our study also identified several other non-visual characteristics associated with driving habits. Depression was associated with driving cessation and may be both a risk factor for and a consequence of driving cessation [14]. Females were more likely to limit their driving [14,16,17]. Females, individuals living with another driver, older individuals, and individuals with impaired cognition were also more likely to prefer another driver. These findings highlight the need to account for non-visual factors when making recommendations regarding driving.

The present study also sought to determine which measure of vision best predicts driving limitations in individuals with glaucoma. Contrast sensitivity appeared to best predict driving cessation, so when information on contrast sensitivity is available, it may be useful in guiding conversations about driving. Contrast sensitivity is highly correlated with better-eye VF MD in glaucoma patients, however so the two measurements are essentially interchangeable for this purpose.

The use of self-report to assess driving habits in this study is a potential source of bias, as subjects may feel motivated to conceal unsafe driving behaviors. It can provide valuable information about subjects’ perceived limitations, however, which may have a strong impact on quality of life. Other studies have used simulators and direct on-road evaluation to assess the impact of glaucoma on driving [7,26-28]. These methodologies have the advantage of allowing direct observation of driving performance, thereby limiting self-report bias, but offer a limited period of observation. Also, the driver is not in their own car or typical driving environment, which may impact their driving performance. The Salisbury Eye Evaluation Driving Study used driver monitoring systems installed in subjects’ cars to study the impact of vision on driving in a population-based cohort of older adults [8]. While that study was not powered to detect the impact of glaucoma on driving, it represents a promising strategy for studying the impact of vision loss on driving in the person’s native environment.

A limitation of our study was that driving changes were not assessed prospectively, so we cannot draw conclusions about the stage of disease at which driving limitations and driving cessation first occurred. Also, our control population was comprised of glaucoma suspects rather than true ‘normals’ not under ophthalmologic care. We felt that this was the best control group for our study as it would balance any potential bias caused by recruitment from a referral center. Additionally, normals recruited from spouses or volunteers are likely to exclude those with mobility limitations, thus overestimating findings. This suspect group reported minimal driving limitations (6.9% driving cessation and an average of 1.1 driving limitations vs. 22.5% cessation and 1.9 limitations for the glaucoma group), suggesting that these glaucoma suspects were not altering their driving habits due to knowledge of their disease risk. Finally, we utilized better-eye VF loss as a metric of glaucoma severity instead of measures aimed at integrating right and left eye VF results to simulate binocular VF loss. Previous work has shown that better-eye MD is typically slightly worse than integrated VF MD, and differs from integrated VF MD by 2 dB or more in roughly one in 4 patients [29,30]. Thus, it is possible that results would have been different if integrated VF MD was used as a metric of VF loss. However, binocular VF loss is not easily calculated in clinic and did not predict subjective or objective measures of disability better than better-eye VF loss in one study [31]. Better-eye MD has been shown to predict legal fitness to drive in the United Kingdom just as well as models which incorporated VF MDs from both eyes [32], though further work will be necessary to determine if better-eye and integrated VF metrics predict actual disability to a similar extent.

The choice to cease or limit driving is likely guided by both fitness to drive and other factors. Regulating fitness to drive is best achieved by combining information regarding real-world patient choices and observation of driving in simulated or real-world situations, and relating them to measures of VF loss [33]. The fact that patients with glaucoma are more likely to limit their driving is encouraging and may indicate that glaucoma patients as a whole are self-regulating in such a way as to keep themselves (and others) safe on the road. Further prospective study is merited to assess when and why people with glaucoma change their driving habits, and perhaps to assess MVAs per mile driven in this group. Because of the potential for driving cessation and limitation to negatively affect individuals’ quality of life, it will be important to balance safety and independence for drivers with visual impairment due to glaucoma.

## Competing interests

The authors declare that they have no competing interests relating to this work. This research was supported in part by the Dennis W. Jahnigen Memorial Award, NIH Grant EY018595, the Research to Prevent Blindness Robert and Helen Schaub Special Scholar Award, the Intramural Research Program of the NIH (National Institute on Aging), and the Doris Duke Charitable Research Foundation Clinical Research Fellowship. All funding organizations had no role in the design or conduct of this research.

## Authors’ contributions

SV participated in data collection, data analysis, and drafting of the manuscript. CH participated in data collection and data analysis. RM participated in data analysis. EC participated in study design and data collection. DF participated in study design. PR participated in study design, data analysis, and oversaw conduct of the study. All authors contributed to revising the manuscript for important intellectual content, read, and approved the final manuscript.

## Pre-publication history

The pre-publication history for this paper can be accessed here:

http://www.biomedcentral.com/1471-2415/13/4/prepub
